# How to predict postoperative atrial fibrillation after cardiac surgery

**DOI:** 10.1002/joa3.12977

**Published:** 2023-12-13

**Authors:** Naoya Kataoka, Teruhiko Imamura

**Affiliations:** ^1^ Second Department of Internal Medicine University of Toyama Toyama Japan

To editor

The prediction and management of postoperative atrial fibrillation (POAF) remain areas yet to be fully elucidated. Rizza and colleagues recently unveiled a connection between several factors, notably the preoperative neutrophil‐to‐lymphocyte ratio (NLR), and the emergence of subacute POAF in patients undergoing cardiac surgery followed by cardiac rehabilitation.^1^ Nevertheless, certain critical considerations have been raised.

It is imperative to provide more comprehensive baseline characteristics associated with atrial fibrillation (AF) to accurately estimate the risk of AF occurrence and its prognostic impact. Such parameters encompass a patient's history of catheter ablation for AF, the administration of anti‐arrhythmic agents and anticoagulants, as well as the evaluation of the CHADS2 score. Notably, patients with a history of paroxysmal AF are widely recognized as being at an escalated risk of AF recurrence and should ideally be excluded from analyses.

The etiology of POAF is multifaceted, intertwined with postsurgical disruptions to the autonomic and inflammatory systems, cytokine cascades, and oxidative stress.^2^ Intriguingly, the authors highlighted the impact of preoperative NLR.^1^ It would greatly benefit the readership if the authors could elucidate the mechanistic underpinnings behind how preoperative parameters influence the incidence of POAF. Additionally, there exists a cadre of other proposed biomarkers predictive of POAF, such as high‐sensitivity C‐reactive protein, markers related to oxidative stress, and uric acid.^3^

Moreover, there is a pressing need to explore the long‐term clinical outcomes associated with POAF. The recurrence of AF frequently manifests in patients who have experienced POAF, thereby influencing mortality and morbidity.^4^

## CONFLICT OF INTEREST STATEMENT

Authors declare no conflict of interests for this article.

## References

[1] Rizza V , Maranta F , Cianfanelli L , Cartella I , Maisano F , Alfieri O , et al. Subacute postoperative atrial fibrillation after heart surgery: incidence and predictive factors in cardiac rehabilitation. J Arrhythm. 2023. 10.1002/joa3.12956 PMC10848578 38333376

[2] Dobrev D , Aguilar M , Heijman J , Guichard JB , Nattel S . Postoperative atrial fibrillation: mechanisms, manifestations and management. Nat Rev Cardiol. 2019;16:417–436. 30792496 10.1038/s41569-019-0166-5

[3] Koi T , Kataoka N , Uchida K , Imamura T , Kinugawa K . Urinary isoxanthopterin as a novel predictor following catheter ablation for atrial fibrillation. J Arrhythm. 2023;39:159–165. 37021030 10.1002/joa3.12828 PMC10068925

[4] Abdelmoneim SS , Rosenberg E , Meykler M , Patel B , Reddy B , Ho J , et al. The incidence and natural progression of new‐onset postoperative atrial fibrillation. JACC Clin Electrophysiol. 2021;7:1134–1144. 33933413 10.1016/j.jacep.2021.02.005

